# *Veillonella* Bacteremia in Alcoholic Hepatitis

**DOI:** 10.1155/2021/9947213

**Published:** 2021-10-13

**Authors:** Patrick Lee, Brandon K. K. Fields, Tom Liang, Michael P. Dubé, Seth Politano

**Affiliations:** ^1^Keck School of Medicine, University of Southern California, Los Angeles, CA 90033, USA; ^2^Department of Medicine, University of Southern California, Los Angeles, CA 90033, USA; ^3^Scripps Mercy Hospital San Diego, San Diego, CA 92103, USA; ^4^Department of Pathology, University of Southern California, Los Angeles, CA 90033, USA; ^5^Department of Infectious Diseases, University of Southern California, Los Angeles, CA 90033, USA

## Abstract

*Veillonella* species are commensal bacteria of the human oral, gut, and vaginal microbiota that are rarely identified as clinically relevant pathogens. Here, we describe a novel case of *Veillonella atypica* bacteremia in a patient with biopsy-proven alcoholic hepatitis. *Veillonella* species have been correlated with disease severity and hepatic encephalopathy in liver diseases such as autoimmune hepatitis and cirrhosis. Their abundance has also been recently observed to be increased in alcoholic hepatitis, where postinflammatory infections are known to impact mortality. This case report highlights the possible clinical manifestations that result from significant gut dysbiosis in patients with severe alcoholic hepatitis. Early identification and treatment of *Veillonella* bacteremia in susceptible populations could be crucial to survival given this organism's predilection for causing life-threatening infections, including meningitis, endocarditis, and osteomyelitis.

## 1. Introduction

*Veillonella* species is an anaerobic, Gram-negative diplococci found in the human oral, gastrointestinal, and vaginal microbiota. Although *Veillonella* is considered a commensal, it has rarely been isolated as a clinically significant pathogen in cases of osteomyelitis, endocarditis, epidural abscess, meningitis, pyelonephritis, and bacteremia [1, 2]. In this case report, we present a case of *Veillonella atypica* bacteremia without a readily identifiable source in the setting of alcoholic hepatitis. To the best of our knowledge, there have only been two previously reported cases of *Veillonella* bacteremia without a clear source of infection [2, 3].

## 2. Case Presentation

A 31-year-old male with a history of heavy alcohol use presented to our hospital with a 3-day history of lower extremity edema, abdominal swelling, and periumbilical pain. He reported use of 6 cans of beer daily, with the most recent use 3 days prior to admission. On presentation, the patient was alert and oriented only to person and self. His vital signs were a temperature of 38.5°C, heart rate 129 beats/minute, and blood pressure 132/74 mmHg. Physical exam was significant for scleral icterus, asterixis, systolic murmur, abdominal distention with caput medusae, palmar erythema, and spider angiomas on his chest.

Laboratory tests revealed a white blood cell (WBC) count of 11.7 K/mm^3^ (with 78% neutrophils, absolute lymphocyte count 1.3 K/mm^3^, and absolute monocyte count 1.0 K/mm^3^), platelets 88 K/mm^3^, lactate 2.1 mmol/L, alkaline phosphatase (ALP) 503 U/L, aspartate aminotransferase (AST) 172 U/L, and alanine aminotransferase (ALT) 19 U/L. Maddrey's Discriminant Function score was 35.3 (Prothrombin time 19.2 seconds and total bilirubin 6.8 mg/dL). Ascitic fluid analysis after paracentesis was negative for spontaneous bacterial peritonitis, with a nucleated cell count of 79/cumm and 23% segmented neutrophils. Further hepatitis workup yielded antinuclear antibodies positive for nuclear, speckled pattern with 1 : 1280 titer, actin antibodies IgG 57 units, and negative viral hepatitis panel for hepatitis A, B, and C. Abdominal ultrasound showed cirrhosis with hepatosplenomegaly and trace abdominal ascites.

**Figure 1:**
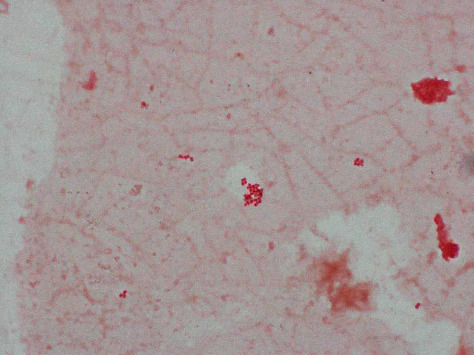
Gram stain from the anaerobic culture plate showing small Gram-negative diplococci (1000x).

**Figure 2:**
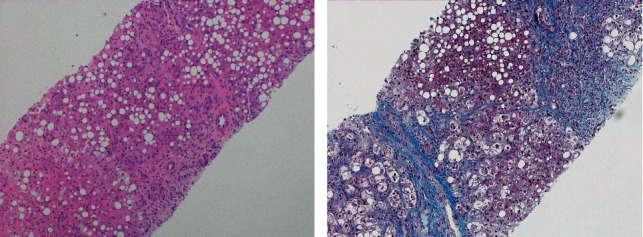
(a) Liver biopsy showing portal and perivenular fibrosis with severe bridging fibrosis and alcoholic hepatitis (hematoxylin & eosin stain, 200x). (b) Trichrome stain demonstrating severe fibrosis (200x).

Two sets of blood cultures obtained on hospital day 0 were negative. Intravenous ceftriaxone was started after collection of the initial blood cultures. Two additional anaerobic blood culture bottles obtained on day 2 of hospital stay were positive for *Veillonella atypica*, identified using MALDI-TOF (Figure 1). Repeat blood cultures after 2 days of intravenous ceftriaxone were negative. The infectious disease team recommended discontinuing IV ceftriaxone as repeat cultures were negative and the patient had completed a 7-day course of ceftriaxone. Transthoracic echocardiogram was negative for valvular lesions. Liver biopsy obtained on hospital day 8 demonstrated bridging fibrosis and was consistent with alcoholic hepatitis with cholestasis (Figures 2(a) and 2(b)).

Hospital course was complicated by hemodynamic instability and acute encephalopathy on day 3 and 4, where patient was tachycardic to 120 beats per minute, tachypneic to 30 shallow breaths per second, and febrile to 38.3°C. The patient's mental status was waxing and waning; there was a concern for sepsis, grade II hepatic encephalopathy, and alcohol withdrawal. Hemodynamic instability and mental status improved with IV lorazepam and lactulose, and the patient was subsequently discharged on hospital day 9. After confirmation of alcoholic hepatitis on liver biopsy, the patient started prednisolone 40 mg oral daily on day 7 after hospital discharge due to difficulty obtaining the prescription.

Patient was evaluated in outpatient hepatology clinic 7 days after prednisolone therapy (14 days after hospital discharge). Pertinent labs showed a white blood cell count of 23.9 K/mm^3^, sodium of 123 mmol/L, and potassium of 2.9 mmol/L. Prothrombin time and total bilirubin had increased to 23.4 sec and 20.2 mg/dL, respectively. Calculated Lille score on day 7 of treatment was 0.577 and suggestive of treatment failure of alcoholic hepatitis. The patient was sent to the emergency room after the clinic visit for tachycardia and concern for sepsis. He was then readmitted to the hospital for further management. Repeat blood cultures and ascitic fluid culture on admission were negative. The patient was deemed a poor transplant candidate due to continued alcohol use and non-United States citizen status. After appropriate counseling, he left the hospital against medical advice to be home with family.

During a phone follow-up 3 months after discharge, the medical team was informed that the patient was deceased.

## 3. Discussion

In this 31-year-old male with alcoholic hepatitis, his hospital course was complicated by sepsis due to *Veillonella atypica* bacteremia. In patients with severe alcoholic hepatitis, the rates of infection range from 12 to 26% of patients, with the most common bacteria cultured being *Escherichia coli* [4]. Timing of infection was noted to be important. Infections present at admission did not impact mortality, but infections that occurred during hospitalization were associated with worse outcomes [4]. The pathophysiology of bacteremia in the setting of alcoholic hepatitis is thought to involve increased intestinal permeability secondary to the proinflammatory state, making the host more susceptible to bacterial translocation. This can result in bacteremia, with subsequent systemic inflammatory response syndrome [4].

It should be noted that although *Veillonella* is rarely a clinically significant pathogen, it has been reported in serious, potentially fatal infections, such as meningitis, pulmonary infections, endocarditis, and osteomyelitis [1, 2, 5]. However, no case reports have observed *Veillonella* bacteremia in the setting of alcoholic hepatitis. Bacterial isolates of *Veillonella atypica* from blood cultures taken on hospital day 2 were sensitive to ampicillin/sulbactam, clindamycin, imipenem, meropenem, and metronidazole and notably resistant to penicillin. Although the treatment for *Veillonella* bacteremia is not well defined, the bacteremia cleared with intravenous ceftriaxone treatment [6]. Previous case reports have discussed use of beta-lactams (notably penicillin, ampicillin, and first-generation cephalosporins), aminoglycosides, metronidazole, and clindamycin in the treatment of *Veillonella* bacteremia in the setting of endocarditis [7]. To date, there is no clear consensus on the treatment for *Veillonella* infections given the limited reports on its pathogenicity [5].

*Veillonella* abundance has been observed to be increased in patients with cirrhosis, autoimmune hepatitis, primary biliary cirrhosis [8], primary sclerosing cholangitis [9, 10], and alcoholic hepatitis [11]. In autoimmune hepatitis, *Veillonella dispar* was identified to be the species most significantly associated with severity of disease, correlated with serum AST [12]. *Veillonella* abundance was also found to be associated with increased systemic inflammation, endotoxemia, and more pronounced in patients with hepatic encephalopathy [13–15]. A recent study by Kim et al. observed significant gut dysbiosis, defined as a decrease in bacterial diversity and autochthonous bacteria levels, and increased *Veillonella* abundance in patients with severe alcoholic hepatitis [11]. Treatment with rifaximin in these patients improved gut dysbiosis and caused a decrease in *Veillonella* abundance [11]. Rifaximin has also been shown to decrease *Veillonella* abundance, improve endotoxemia, and improve intestinal barrier function in patients with cirrhosis complicated by hepatic encephalopathy [13]. The unique presentation of *Veillonella* bacteremia in this patient likely reflects the significant gut dysbiosis in patients with severe alcoholic hepatitis.

The role of the gut microbiota, consisting of archaea, fungi, viruses, protozoans, and bacteria such as *Veillonella*, has been described in human diseases, including obesity, diabetes, and cardiovascular diseases [16]. Recent research has demonstrated its association in liver diseases as well. Studies have observed unique gut microbiota compositions in autoimmune liver disease [8], nonalcoholic fatty liver disease (NAFLD) [17], and cirrhosis [14, 15]. Interestingly, the gut microbiota composition shifts with severity of liver disease, such as worsening fibrosis in NAFLD [17, 18]. Besides medications, such as the antibiotic rifaximin, probiotics and fecal microbiota transplant (FMT) have been investigated as targeted therapies to alter the gut microbiota to treat certain human diseases. Recent studies have demonstrated significant improvements in survival in patients with steroid-ineligible alcoholic hepatitis treated with FMT, suggesting that FMT can be a cost-effective transition to liver transplant or augment transplant-free survival in these patients [19, 20]. Probiotics and FMT have also been shown to improve cognitive function in patients with cirrhosis complicated by hepatic encephalopathy [21, 22]. These recent studies highlight the potential for targeted therapies towards the gut microbiota to treat complications of conditions such as alcoholic hepatitis and hepatic encephalopathy.

In summary, this patient passed away from alcoholic hepatitis that was nonresponsive to steroid therapy. A Lille model score >0.45 predicts a 6-month survival of only 25% [23]. The positive results in the studies of FMT in alcoholic hepatitis demonstrate a potential alternative or adjunct to steroid therapy [19, 20]. There remains a great need for further research into alcoholic hepatitis given its high mortality, and novel therapeutic options involving immunomodulation are currently being investigated [24].

## Data Availability

The data used to support the findings of this study are included within the article.
